# Andrographolide suppresses the migratory ability of human glioblastoma multiforme cells by targeting ERK1/2-mediated matrix metalloproteinase-2 expression

**DOI:** 10.18632/oncotarget.22407

**Published:** 2017-11-11

**Authors:** Shih-Liang Yang, Fu-Hsuan Kuo, Pei-Ni Chen, Yi-Hsien Hsieh, Nuo-Yi Yu, Wei-En Yang, Ming-Ju Hsieh, Shun-Fa Yang

**Affiliations:** ^1^ Institute of Medicine, Chung Shan Medical University, Taichung, Taiwan; ^2^ Department of Traditional Chinese Medicine, Taichung Hospital, Ministry of Health and Welfare, Taichung, Taiwan; ^3^ Neurological Institute, Taichung Veterans General Hospital, Taichung, Taiwan; ^4^ Institute of Biochemistry, Microbiology and Immunology, Chung Shan Medical University, Taichung, Taiwan; ^5^ Clinical Laboratory, Chung Shan Medical University Hospital, Taichung, Taiwan; ^6^ Department of Medical Research, Chung Shan Medical University Hospital, Taichung, Taiwan; ^7^ Cancer Research Center, Changhua Christian Hospital, Changhua, Taiwan; ^8^ Graduate Institute of Biomedical Sciences, China Medical University, Taichung, Taiwan

**Keywords:** glioblastoma multiforme, andrographolide, migration, CREB

## Abstract

Glioblastoma multiforme (GBM) can be a fatal tumor because of difficulties in treating the related metastasis. Andrographolide is the bioactive component of the *Andrographis paniculata*. Andrographolide possesses the anti-inflammatory activity and inhibits the growth of various cancers; however, its effect on GBM cancer motility remains largely unknown. In this study, we examined the antimetastatic properties of andrographolide in human GBM cells. Our results revealed that andrographolide inhibited the invasion and migration abilities of GBM8401 and U251 cells. Furthermore, andrographolide inhibited matrix metalloproteinase (MMP)-2 activity and expression. Real-time PCR and promoter activity assays indicated that andrographolide inhibited MMP-2 expression at the transcriptional level. Such inhibitory effects were associated with the suppression of CREB DNA-binding activity and CREB expression. Mechanistically, andrographolide inhibited the cell motility of GBM8401 cells through the extracellular-regulated kinase (ERK) 1/2 pathway, and the blocking of the ERK 1/2 pathway could reverse MMP-2-mediated cell motility. In conclusion, CREB is a crucial target of andrographolide for suppressing MMP-2-mediated cell motility in GBM cells. Therefore, a combination of andrographolide and an ERK inhibitor might be a good strategy for preventing GBM metastasis.

## INTRODUCTION

Glioblastoma multiforme (GBM) is the most common and most aggressive malignant primary brain tumor in humans [1]. GBM has high proliferation rate and invasiveness, which be treated with surgical extirpation, local irradiation, and conventional chemotherapy with temozolomide (TMZ) [2, 3]. Moreover, in nearly 20% of patients treated with TMZ, significant clinical toxicity is regularly observed [4]. Because of the side effects, chemotherapy with TMZ has limited efficiency. Therefore, it is needed to develop new approaches to the current medical treatment options for glioblastomas.

Poor clinical outcomes in glioblastomas are largely due to their infiltrating nature and recurrence at the adjacent or distant regions of the brain [5]. The metastasis of cancer cells involves several processes including the invasion of the surrounding tissue and the formation of new tumors [6–8]. Breakdown of the ECM is one of the cancer cell metastasis process which mediated by matrix metalloproteinases (MMPs) of various types of human cancers [9–11]. Therefore, the inhibition of migration mediated by MMP-2 or MMP-9 can putatively provide a preventive measure against cancer metastasis [12–16]. MMP-2 and MMP-9 are highly expressed in glioblastomas, and the expression increases with tumor development at both the messenger RNA and protein levels [17–20]. Moreover, numerous studies shown that downregulation of MMP-2 expressions contribute to the inhibition of metastasis in glioblastoma cells [21–23]. However, the mechanisms that regulate MMP-2 gene transcription in human glioma cells are not fully elucidated.

In current years, plant products have gained increasing attention for potential use in interventions against tumor invasive progression in neoplastic diseases [24, 25]. Andrographolide, a diterpenoid lactone isolated from *Andrographis paniculata*, inhibits cancer cell development [26–28] and has potent anti-inflammatory [29–31] and anti-cancer invasion and migration activities [32, 33]. Lee et al. indicated that andrographolide inhibited HMGB1-mediated hyperpermeability and leucocyte migration in septic mice. The results revealed that andrographolide repressed the tumor necrosis factor-α expression via AKT, extracellular-regulated kinase (ERK) 1/2 pathway in human umbilical vein endothelial cells (HUVECs) [34]. However, the anticancer growth effects and migratory or invasive effects of andrographolide on glioblastoma cells have not been investigated yet. Therefore, we hypothesized that andrographolide has an impact on the migration and invasion of glioblastoma cells. In this study, we evaluated the ability of andrographolide to suppress the migration and invasion of glioblastoma cells and elucidated the underlying molecular mechanisms.

## RESULTS

### Cytotoxic effects of andrographolide on human GBM cells

**Figure 1:**
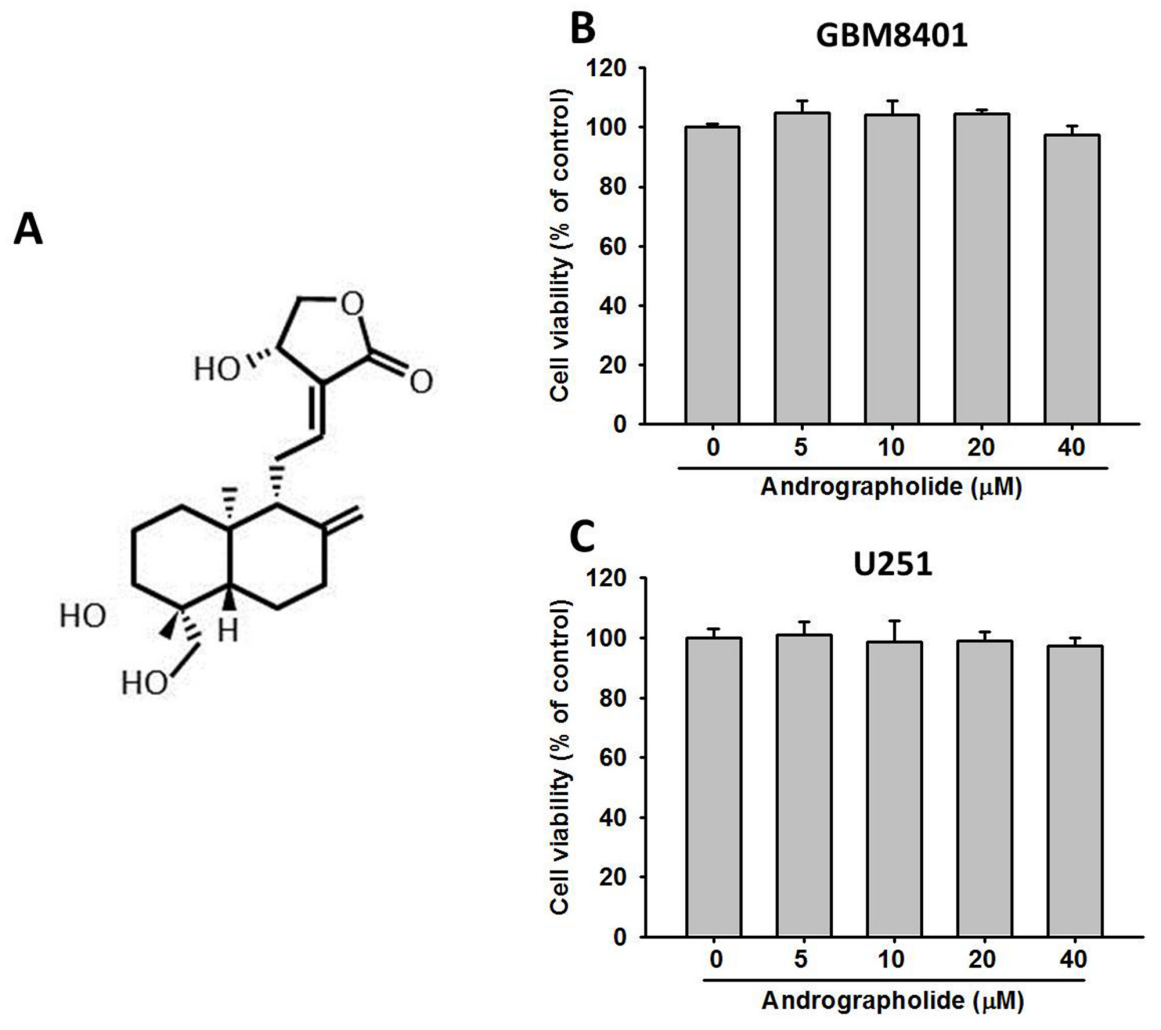
Effects of andrographolide on cell viability **(A)** Structure of andrographolide. Cell viability of **(B)** GBM8401 cells and **(C)** U251 cells cultured in presence of andrographolide (0-40 μM) for 24 h, as analyzed by MTT assay. Results are shown as mean ± SE from 3 determinations per condition repeated 3 times.

The chemical structure of andrographolide is presented in Figure 1A. To assess the effects of andrographolide on cell viability, GBM8401 and U251 cells were treated with andrographolide at various concentrations (0-40 μM) for 24 h and then analyzed using the MTT assay. At the highest concentration of 40 μM, andrographolide did not alter the viability of GBM8401 and U251 cells after treatment for 24 h compared with that of the controls (Figure 1B and 1C). Thus, the andrographolide concentration range of 0 to 40 μM was used in all subsequent anticancer motility experiments.

### Effects of andrographolide on migration and invasion in human GBM cells *in vitro*

**Figure 2:**
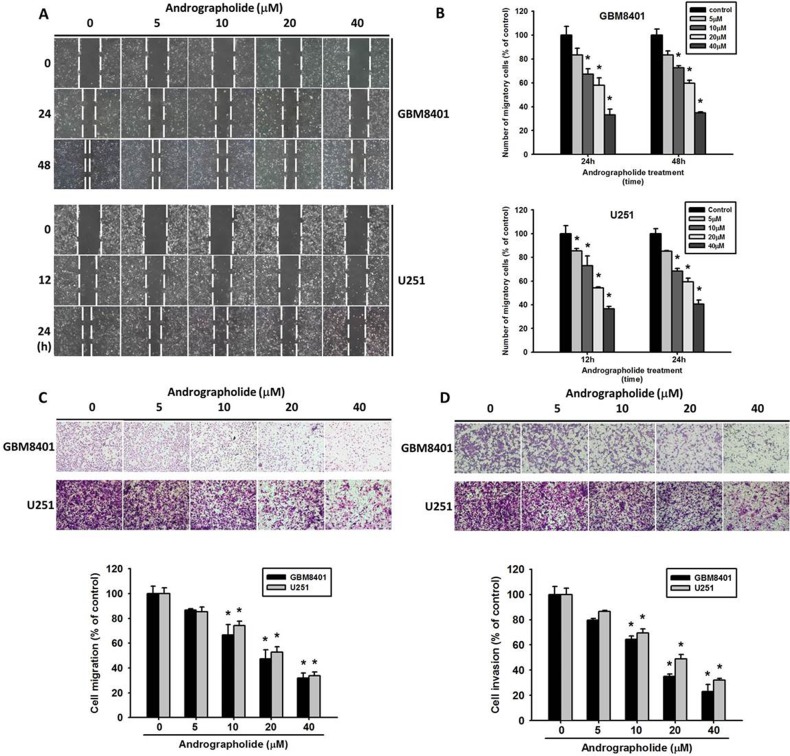
Andrographolide inhibits wound healing assay, migration and invasion in the GBM8401 and U251 cell lines **(A-B)** GBM8401 and U251 cells were wounded and then treated with andrographolide (0-40 μM) for 24 h in a serum-containing medium. At 0, 24 and 48 h (for GBM8401) or at 0, 12 and 24 h (for U251 cell), phase-contrast pictures of the wounds at four different locations were taken. **(C-D)** Human GBM8401 and U251 cells were treated with andrographolide (0-40 μM); cell migration and cell invasion was subsequently measured using a Boyden chamber and a Matrigel-coated Boyden chamber as described in material and methods section. ^*^Significantly different, p < 0.05, compared with the vehicle group.

The effect of andrographolide on the cell migration ability of GBM8401 and U251 cells was investigated. Figure 2A shows representative photographs of the migration of GBM8401 and U251 cells. The number of migrated cells decreased in a concentration-dependent manner (Figure 2B). At 40 μM, andrographolide reduced the number of migrated GBM8401 cells by 68% at 48 h. Figure 2C and 2D illustrate the effects of andrographolide on cell migration and cell invasion in GBM8401 and U251 cells, respectively. At 40 μM, andrographolide reduced GBM8401 cell invasion by 74% (Figure 2D). The results indicate that andrographolide markedly reduced the migration and invasion abilities of GBM8401 and U251 cells in a dose-dependent manner.

### Andrographolide inhibits the activity and protein expression of MMP-2 in GBM cells

**Figure 3:**
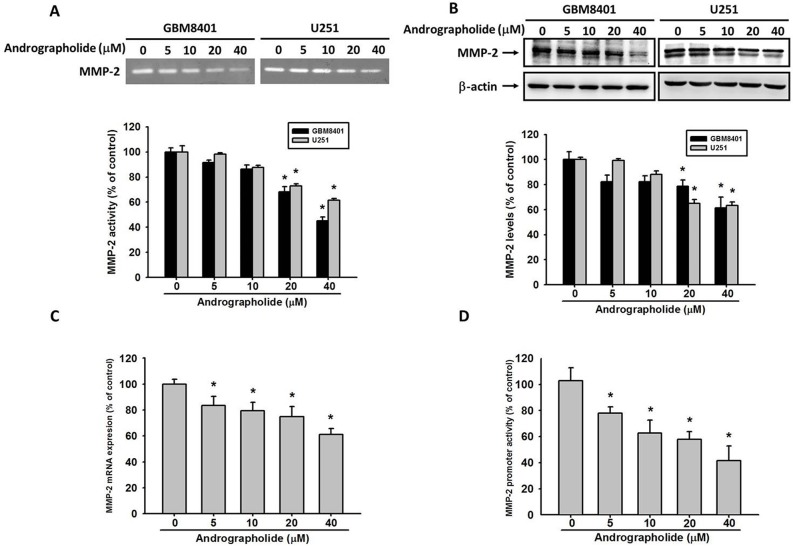
Andrographolide inhibit MMP-2 activity, protein and mRNA expression **(A)** GBM8401 cells and U251 cells were treated with andrographolide (0-40 μM) for 24 h in serum free medium and then subjected to gelatin zymography to analyze the activity of MMP-2. **(B)** Western blotting to analyze the protein levels of MMP-2. Quantitative results of MMP-2 protein levels after being adjusted with β-actin. The values represented the means ± SE from 3 determinations per condition repeated 3 times. **(C)** GBM8401 cells were treated with andrographolide (0-40 μM) for 24 h and then subjected to real-time PCR to analyze the mRNA expression of MMP-2. **(D)** MMP-2 promoter reporter assay to analyze the promoter activity of MMP-2. Luciferase activity, determined in triplicates, was normalized to β-galactosidase activity. The values represented the means ± SE from 3 determinations per condition repeated 3 times (n=3). ^*^Significantly different, p < 0.05, compared with the vehicle group.

MMP-2 is a protease involved in the degradation of ECM in tumor metastasis [35, 36]. To investigative the mechanisms through which andrographolide inhibits cell invasion and migration in GBM cells, we analyzed the expression levels of MMP-2. The results revealed that treatment with andrographolide (40 μM) significantly inhibited MMP-2 enzyme activity (Figure 3A) and protein expression (Figure 3B) in both GBM8401 and U251 cells.

### Andrographolide suppresses MMP-2 expression at the transcriptional level

To investigate the inhibitory effects of andrographolide on MMP-2 transcriptional level in GBM8401 cells, the cells were treated with andrographolide at various concentrations (0-40 μM) for 24 h, and mRNA levels were then analyzed through real-time PCR. The results demonstrated that MMP-2 mRNA levels significantly decreased (Figure 3C). The results of the promoter analysis performed using a luciferase assay kit indicated that andrographolide significantly inhibits the luciferase activities of MMP-2 (Figure 3D). These results indicate that andrographolide regulates the MMP-2 expression at the transcriptional level in GBM8401 cells.

### CREB is the key regulator for the transcriptional inhibition of MMP-2 by andrographolide

**Figure 4:**
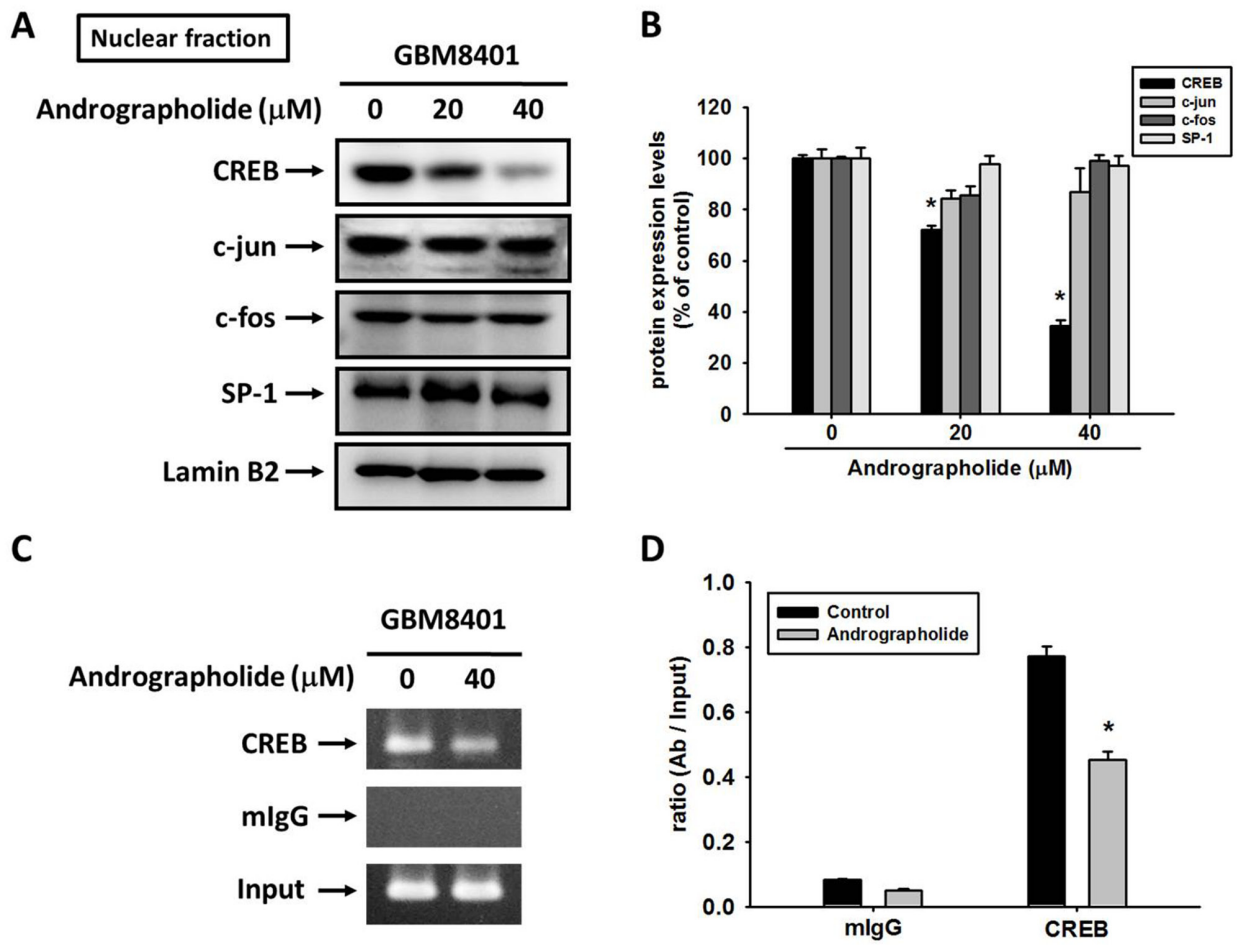
Critical role of CREB in andrographolide-induced transcriptional inhibition of MMP-2 in GBM8401 cells **(A-B)** GBM8401 cells were treated with andrographolide (0-40 μM) for 24 h and then the nuclear fraction was prepared as described in Materials and Methods. Representative results of CREB, c-jun, c-fos and SP-1 by Western blot analysis. **(C-D)** ChIP analysis of the association of CREB transcription factors with the MMP-2 promoter region in GBM8401 cells. The values represented the means ± SE from 3 determinations per condition repeated 3 times (n=3). ^*^Significantly different, p < 0.05, compared with the vehicle group.

The sequence analysis of the MMP-2 promoter indicated numerous cis-acting regulatory elements, including CREB, SP-1, and AP-1 that could be involved in the regulation of MMP-2 expression [37, 38]. To examine whether specific transcription factors are involved in the transcriptional inhibition of MMP-2 by andrographolide in GBM8401 cells, we evaluated the effect of andrographolide on the nuclear translocation of CREB, SP-1, c-fos, and c-Jun. The treatment of GBM8401 cells with andrographolide (0, 20, and 40 μM) reduced the nuclear translocation of CREB, but not that of SP-1, c-Jun, or c-fos, in a concentration-dependent manner (Figure 4A and 4B). We further performed a ChIP assay to investigate the involvement of CREB transcription factors in the transcriptional inhibitory effects of andrographolide on MMP-2. The binding of CREB to the MMP-2 promoter decreased in GBM8401 cells after treatment with andrographolide (Figure 4C and 4D). These findings indicate that andrographolide causes the transcriptional inhibition of MMP-2 in GBM8401 cells by suppressing the nuclear translocation of CREB and the binding activity of the MMP-2 promoter.

### Role of the ERK1/2 pathway in the andrographolide-mediated inhibition of cell motility and MMP-2 expression

**Figure 5:**
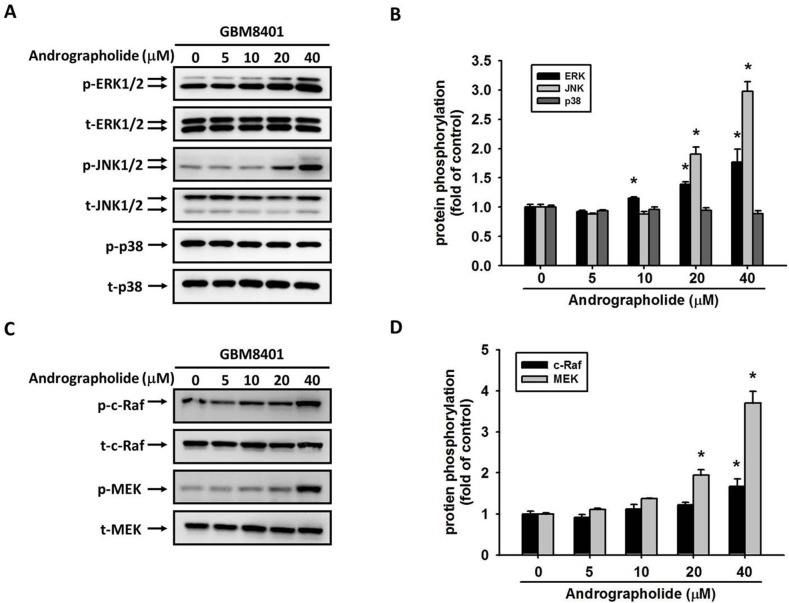
Effect of andrographolide on MAPK pathway in GBM8401 cells GBM8401 cells were treated with andrographolide (0-40 μM) for 24 h and then the cell lysates were subjected to SDS-PAGE followed by western blots with **(A)** anti-ERK1/2, anti-JNK, anti-p38, **(C)** anti-c-Raf and anti-MEK antibodies as described in Materials and Methods. **(B, D)** Quantitative results the phosphorylation levels of ERK1/2, JNK, p38, c-Raf and MEK. ^*^Significantly different, p < 0.05, compared with the vehicle group.

**Figure 6:**
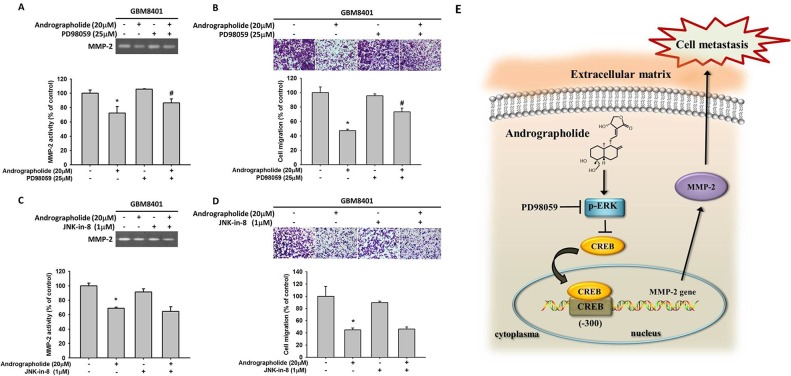
Effects of MEK inhibitor (PD98059), JNK inhibitor (JNK-in-8) and andrographolide on MMP-2 activity and cell migration of GBM8401 cells GBM8401 cells were pre-treated with PD98059 (25 μM) for 30 min, and then incubated in the presence or absence of andrographolide (20 μM) for 24 h. **(A)** The culture media were used as subjects for analysis of MMP-2 activity. **(B)** The cells were used for migration assay as described in the Materials and Methods section. **(C-D)** GBM8401 cells were pre-treated with JNK-in-8 (1 μM) for 30 min, and then incubated in the presence or absence of andrographolide (20 μM) for 24 h. (C) The culture media were used as subjects for analysis of MMP-2 activity. (D) The cells were used for migration assay as described in the Materials and Methods section. The values represented the means ± SD of at least three independent experiments. ^*^p<0.05 as compared with the control. #Significantly different, p<0.05, when compared with andrographolide-treated group. **(E)** A working model showing the ability of andrographolide to regulate the invasive ability of GBM8401 cells.

Studies have reported that the activation of ERK, a key molecule located in the MAPK pathway, is responsible for the activation of MMP-2 [39] and the invasion of cancer cells including GBM cells [21]. Therefore, we determined whether MAPK pathway plays a role in the andrographolide-mediated suppression of cell motility and MMP-2 expression in GBM8401 cells. The results revealed that andrographolide enhanced the phosphorylation of ERK 1/2 and JNK 1/2, whereas the phosphorylation of p38 was not changed in GBM8401 cells (Figure 5A and 5B). To further define the signaling pathway that mediates ERK activation, we determined whether upstream activators, c-Raf and MEK, are also activated by andrographolide. As shown in Figure 5C and 5D, the phosphorylation of c-Raf and MEK was also increased after andrographolide treatment in GBM8401 cells. Next, we investigated relationships among the andrographolide-mediated inhibition of MMP-2, cell motility, and JNK and ERK activation. The pretreatment of GBM8401 cells with a highly specific inhibitor of MEK (PD98059) significantly reversed MMP-2 activity (Figure 6A) and cell migration (Figure 6B); however, the JNK inhibitor (JNK-in-8) did not alter the MMP-2 activity and cell migration (Figure 6C and 6D). Taken together, these results indicate that the c-Raf/MEK/ERK pathway might play a role in regulating the motility of GBM cells.

## DISCUSSION

The causes of death of patients with glioma are the deep occurrence of gliomas in the brain and the highly proliferative properties of gliomas. Therefore, early prevention of glioma growth by chemopreventive agents may be warranted. Extensive studies have indicated that the natural products may arrest tumor promotion and progression in various human cancer cell lines by controlling cell apoptosis or invasion. Flavonoids widely exist in vegetables, fruits and red wine and humans consume approximately 1 g of flavonoids in their diet daily. Numerous beneficial biological properties of flavonoids, including anti-inflammatory, anti-oxidant, and anti-tumor activities, have been identified [13, 36, 37, 40–43], and some types of flavonoids have been reported to exert therapeutic effects on brain diseases [44] including brain tumors [21]. Andrographolide, the major constituent of the *A. paniculata* extract, is involved in the anticancer activity [45]. However, no studies have described the antimetastasis (inhibition of invasion and migration) effects of GBM cells. To the best of our knowledge, this is the first study to demonstrate that andrographolide inhibits the migration and invasion of GBM cells.

Invasion by glioma cells is a multistep process involving degradation of ECM components, and subsequent infiltration into adjacent brain tissues. This process is largely attributable to the activation of MMPs. MMPs play vital roles in tumor angiogenesis and metastasis [10, 46]. The inhibition of MMP-2 and MMP-9 enzyme activities can prevent of cancer metastasis [47–49]. Moreover, glioma cells express various MMPs, among which MMP-2 is supposed to most effectively degrade ECM components [50–52]. Similarly, our study results revealed that MMP-2 was highly secreted by GBM cells, and overexpression of MMP-2 has been found in clinical specimens and to be correlated with tumor invasion in gliomas [10, 36]. Moreover, from a search of available microarray data (PrognoScan database), our previously study observed that MMP-2 has been negatively correlated with the overall survival rate of patients with glioma [21]. These findings indicate that MMP-2 might be a crucial regulator of tumor metastasis in GBM. The results of the present study indicated that andrographolide significantly inhibited MMP-2 promoter activity, mRNA level and protein expression in GBM8401 cells (Figure 3). The results indicating that andrographolide inhibits the MMP-2 expression at the transcriptional level.

Several regulatory elements, including p53, AP-1, CREB, SP-1, and AP-2, which could be involved in regulating MMP-2 expression [37, 38]. Our study indicated that the regulation of MMP-2 by andrographolide occurred at the transcriptional level and was mainly mediated by CREB. The transcriptional activity of CREB plays a crucial role in tumor metastasis in several cancer cell types including GBM [15, 53]. CREB is a ubiquitously expressed transcription factor and is phosphorylated at Ser^133^ by cAMP-dependent protein kinase A and other kinases [54]. It subsequently increases its transcriptional activity by changing its association with CBP/p300 histone acetylase. Our findings implicating that regulation of CREB in the MMP-2 are consistent with those of previous studies on melanomas [55] and ovarian cancer [56]. In addition, we observed that andrographolide can attenuate the DNA-binding activity of CREB in the MMP-2 promoter region.

MAPK pathway is involved in numerous cellular programs, such as cell differentiation, cell death and cell migration [57, 58]. A previous study showed that andrographolide inhibited cell metastasis by interfering with PI3K/Akt and ERK1/2 signaling pathways [59]. Wong et al. also reported that andrographolide induces heme oxygenase 1 in astrocytes by activating ERK1/2 and p38 pathway [60]. Moreover, andrographolide has been reported as a promising anticancer agent that inhibits tumor metastasis [61]. Pratheeshkumar et al. demonstrated that andrographolide inhibits the nuclear translocation of NF-κB and CREB in B16F-10 melanoma cells [62]. Cheng et al. reported that caffeine reduced the invasion of glioma cells through FAK/ERK signaling pathway [63]. As presented in Figure 6, andrographolide enhanced the phosphorylation of the c-Raf/MEK/ERK pathway in GBM8401 cells. To further investigate the related effects of andrographolide on GBM8401 cells, we investigated the effect of andrographolide combined with a specific inhibitor of the MEK pathway (PD98059) on cell migration. We observed that the combined treatment of andrographolide and the aforementioned pathway inhibitor reduced MMP-2 activity and migration. This is the first report that the antimetastasis effect of andrographolide on GBM cells. However, limitation of current *in vitro* study was the lack of *in vivo* animal study, which could provide more support to our current findings and will be included in our future work.

In conclusion, the study demonstrated that andrographolide can inhibit the expression of CREB-DNA binding activity, MMP-2 expression and the inhibition of migration (Figure 6E). Andrographolide also inhibits cell migration by increasing the phosphorylation of the ERK pathway. Thus, inhibition of cancer metastasis by andrographolide can provide crucial therapeutic protection against GBM.

## MATERIALS AND METHODS

### Cell lines

GBM8401 cells were originally isolated and established from an ethnic Chinese female patient with GBM [64]. In this study, human GBM8401 and U251 cell lines were purchased from the Food Industry Research and Development Institute (Hsinchu, Taiwan). GBM8401 and U251 cells were cultured in RPMI 1640 medium supplemented with 10% fetal bovine serum (FBS), 2 mM L-glutamine, 100 U/mL penicillin, and 100 μg/mL streptomycin at 37°C in a humidified atmosphere containing 5% CO_2_.

### Cell viability assay

To determinate cell viability, a colorimetric assay using tetrazolium dye, 3-(4,5-dimethylthiazol-2-yl)-2,5-diphenyltetrazolium bromide (MTT), was performed for evaluating the cytotoxicity of andrographolide (Sigma Chemical Co., St. Louis, MO, USA). GBM8401 and U251 cells (6 × 10^4^ cells/well) were seeded in 24-well plates and treated with the indicated concentrations of andrographolide for 24 h under the same culture condition. The medium was removed after andrographolide treatment. Attached cells were washed with phosphate buffered saline and incubated with 20 μL of 5 mg/mL MTT (Sigma Chemical Co., St. Louis, MO, USA) at 37°C for 4 h. The quantity of viable cells per well was assessed by evaluating the production of formazan, which was measured spectrophotometrically at 563 nm following solubilization with isopropanol.

### *In vitro* wound closure assay

GBM8401 cells (2 × 10^5^ cells/well) and U251 cells (2 × 10^5^ cells/well) were plated in six-well plates for 24 h. The cells were scratched with a pipette tip to wound them, incubated in RPMI medium containing 0.5% FBS, and treated with or without andrographolide (0-40 μM) for 12, 24, or 48 h. The cells were photographed using a phase-contrast microscope (×100), as described previously [65].

### Cell migration and cell invasion assays

Boyden chamber cell migration and invasion assay were assayed according to previously described methods [66]. Briefly, after treatment with andrographolide (0-40 μM) for 24 h, cells were seeded in a Boyden chamber (Neuro Probe, Cabin John, MD) for the invasion and migration assay. For the invasion assay, polycarbonate membrane filters with an 8-μm pore size were precoated with 10 μL of Matrigel (25 mg/50 mL; BD Biosciences, MA); the bottom chamber contained the standard medium. Invaded cells were fixed and stained with 5% Giemsa. Stained cells in each well were photographed and counted. Triplicate samples were examined, and data are expressed as the average cell number in five fields. The migration assay was performed using the procedure described for the invasion assay but without Matrigel coating [38].

### Gelatin zymography

MMP-2 activity in the conditioned medium from GBM8401 cells was measured using gelatin zymography protease assays, as described previously [67]. An appropriate volume of collected media was subjected to electrophoresis on 8% sodium dodecylsulfate-polyacrylamide gel electrophoresis (SDS-PAGE) gel containing 0.1% gelatin. After electrophoresis, the gel was washed with 2.5% Triton X-100 and incubated in a reaction buffer (40 mM Tris-HCl at pH 8.0, 10 mM CaCl_2_, and 0.01% NaN_3_) for 12 h at 37°C. The gel was then stained with Coomassie brilliant blue R-250.

### RNA preparation and TaqMan real-time quantitative PCR

Total RNA was isolated from GBM cells by using Trizol (Life Technologies, Grand Island, NY) according to the manufacturer's instructions. A real-time quantitative (q) PCR analysis was conducted using the TaqMan one-step PCR Master Mix (Applied Biosystems, CA, USA). Total cDNA (100 ng) was added to each 25 μL reaction mix containing MMP-2 or GAPDH primers and TaqMan probes. The MMP-2 (Hs00234422_m1) and GAPDH (Hs99999905_m1) primers and probes were designed using commercial software (ABI PRISM For Peer Sequence Detection System; Applied Biosystems, CA, USA). Real-time qPCR assays were performed in triplicate on a StepOnePlus sequence detection system. The threshold was set above the nontemplate control background and within the linear phase of target gene amplification to calculate the cycle number at which the transcript was detected.

### Western blot analysis

Total cell lysates were prepared and cytosolic proteins were extracted as previously described [68]. Equal amounts of protein extracts were subjected to 10% or 12% SDS-PAGE and blotted onto polyvinylidene fluoride membranes (Millipore, Belford, MA, USA). After blocking, membranes were incubated with primary antibodies. Antibodies, specifically of MMP-2, p-extracellularly regulated kinase (ERK)1/2, p-p38, p-c-Jun N-terminal kinase (JNK), ERK1/2, p38, JNK1/2, and β-actin were purchased from Cell Signaling Technology (Danvers, MA, USA). CREB, C-Jun, c-fos, and SP-1 antibodies were purchased from Santa Cruz Biotechnology. Lamin-B2 antibodies were purchased from GeneTex International Corporation. Blots were then incubated with a horseradish peroxidase (HRP)-conjugated anti-mouse or anti-rabbit antibody. Signals were detected through ECL by using the Immobilon Western HRP Substrate (Millipore, Billerica, MA, USA).

### Nuclear protein extraction

To extract nuclear proteins, protein extracts were prepared from andrographolide-treated GBM8401 cells by using the NE-PER Cytoplasmic and Nuclear Protein extraction kit (Pierce Biotechnology, Rockford, IL, USA).

### Transfection and MMP-2 promoter-driven luciferase assays

GBM8401 cells were seeded at a concentration of 5 × 10^4^ cells/well in six-well cell culture plates. After 24 h of incubation, the cells were cotransfected with pGL3-basic (vector), pMMP-2-luciferase (Luc), and a β-galactosidase expression vector (pCH110) by using Turbofect (Fermentas, Carlsbad, CA). After 12 h of transfection, the cells were treated with a vehicle or andrographolide for 24 h. Cell lysates were harvested, and luciferase activity was determined using a luciferase assay kit. The value of the luciferase activity was normalized to the transfection efficiency and monitored by β-galactosidase expression.

### Chromatin immunoprecipitation analysis

A chromatin immunoprecipitation (ChIP) analysis was performed as described previously [13, 16]. Briefly, GBM8401 cells were washed with PBS and were then treated with 1% formaldehyde to cross-link protein-protein and protein-DNA complexes. After cells were lysed using lysis buffer, DNA immunoprecipitated with antibodies specific to CREB or the control, rabbit immunoglobulin G, was purified and extracted as described previously [13, 16].

### Statistical analysis

The statistical analysis was performed using Statistical Package for the Social Sciences software, Version 16 (SPSS, Chicago, IL). Data were analyzed using Student's t-test when two groups were compared. A p value of <0.05 was considered statistically significant.
